# Association of coronary microvascular dysfunction and cardiac bridge integrator 1, a cardiomyocyte dysfunction biomarker

**DOI:** 10.1002/clc.23726

**Published:** 2021-09-16

**Authors:** Christine Pacheco, Janet Wei, Chrisandra Shufelt, Tara C. Hitzeman, Galen Cook‐Wiens, Carl J. Pepine, Eileen Handberg, R. David Anderson, John Petersen, TingTing Hong, Robin M. Shaw, C. Noel Bairey Merz

**Affiliations:** ^1^ Hôpital Pierre‐Boucher Cardiology Service, Department of Medicine, Université de Montréal Longueuil Quebec Canada; ^2^ Centre Hospitalier de l'Université de Montréal (CHUM), Cardiology Service, Department of Medicine, Université de Montréal Montreal Quebec Canada; ^3^ Barbra Streisand Women's Heart Center Smidt Heart Institute, Cedars‐Sinai Medical Center Los Angeles California USA; ^4^ Smidt Heart Institute Cedars‐Sinai Medical Center Los Angeles California USA; ^5^ Cardiovascular Division, University of Florida Gainesville Florida USA; ^6^ Nora Eccles Harrison Cardiovascular Research and Training Institute University of Utah Salt Lake City Utah USA

**Keywords:** coronary microvascular dysfunction, heart failure, women

## Abstract

**Background:**

Coronary microvascular dysfunction (CMD) is associated with heart failure with preserved ejection fraction (HFpEF); however, pathophysiology is not well described.

**Hypothesis:**

We hypothesized that CMD in women with suspected ischemia with no obstructive coronary artery disease (INOCA) is associated with cardiomyocyte dysfunction reflected by plasma levels of a cardiomyocyte calcium handling protein, cardiac bridge integrator 1 (cBIN1).

**Methods:**

Women with suspected INOCA undergoing coronary function testing were included. Coronary flow reserve, vasodilation to nitroglycerin, change in coronary blood flow (ΔCBF), and vasodilation to acetylcholine (ΔAch) were evaluated. cBIN1 score (CS) levels in these women (n = 39) were compared to women with HFpEF (n = 20), heart failure with reduced ejection fraction (HFrEF) (n = 36), and reference controls (RC) (n = 50). Higher CS indicates cardiomyocyte tubule dysfunction.

**Results:**

INOCA, HFpEF, and HFrEF women were older than RC (*p* < .05). Higher CS was associated with vasoconstriction to acetylcholine (r = −0.43, *p* = .011) with a trend towards lower ΔCBF (r = 0.30, *p* = .086). Higher CS was specific for ΔAch and ΔCBF but had limited sensitivity. INOCA women had higher CS than RC, but lower CS than HFpEF/HFrEF groups (*p* < .001).

**Conclusions:**

CS, a plasma biomarker indicating poor cardiomyocyte health, was higher in women with suspected INOCA as compared to RC, but lower than in women with HFpEF. Elevated CS in suspected INOCA patients represents an intermediate group between health and disease, supporting the hypothesis that CMD may progress to HFpEF. Larger prospective cohort studies are needed to confirm the pathophysiological relationship between cBIN1, CMD, and HFpEF.

## 1 INTRODUCTION

Women with evidence of ischemia and no obstructive coronary artery disease (INOCA) are frequently diagnosed with coronary microvascular dysfunction (CMD) which is associated with an increased risk of adverse events during follow‐up.^1^ The most frequent of these events is heart failure^2^ with preserved ejection fraction (HFpEF);^3^, ^4^ however, underlying mechanisms remain poorly understood. CMD, linked with traditional cardiovascular risk factors^5^ and stressors, through microvascular rarefication and decreased cardiomyocyte energy availability, also has the potential to disrupt mechanistic functioning of cardiomyocytes, including transverse tubules, contributing to increased ventricular remodeling and stiffness.^6^ This may be associated with dysfunctional LV mechanics prior to the onset of overt myocardial dysfunction, fibrosis, and symptoms of heart failure and a circulating biomarker for transverse tubule disruption would be useful for identifying patients with CMD at risk of developing HFpEF.

Cardiac bridge integrator 1 (cBIN1) is a protein distributed along cardiomyocyte t‐tubules organizing calcium releasing dyads responsible for calcium influx and calcium‐induced calcium release, essential for efficient excitation‐contraction coupling and normal contractility.^7^, ^8^, ^9^ Elevated cBIN1 score (CS), a reciprocal dimensionless score derived from plasma cBIN1 levels, is associated with adverse cardiac remodeling,^10^ and has been observed HFpEF^11^ and heart failure with reduced ejection fraction (HFrEF),^12^ however its significance in CMD is unknown. Because both CMD and HFpEF are clinically important conditions among symptomatic women, it would very helpful if these links could be established in a cohort of such women, which could potentially lead to better therapeutic targeting for patients.

Accordingly, we hypothesized that among women with symptoms and signs of suspected INOCA, CMD presence would predict cardiomyocyte dysfunction as reflected by plasma cBIN1 levels.

## 2 METHODS

### 2.1 Women with suspected INOCA

Women sequentially enrolled in the Women's Ischemia Syndrome Evaluation—Coronary Vascular Dysfunction Continuation Study (WISE‐CVD Continuation, ClinicalTrials.gov Identifier: NCT02582021) with suspected INOCA at a single site referred for clinical coronary function testing for evaluation of CMD with CS levels (n = 39) were included between November 1, 2015 and September 1, 2017. Baseline data collected included age, body mass index (BMI) and cardiovascular risk factors. Exclusion criteria included the presence of obstructive coronary artery disease (CAD) (≥50% stenosis), acute coronary syndrome within the last 3 months, chest pain due to a nonischemic etiology, need for valve repair or replacement, patients with cardiogenic shock, left ventricular ejection fraction (LVEF) <50%, previous percutaneous coronary intervention or coronary artery bypass grafting, end‐stage renal or liver disease, life expectancy <4 years, or inability to give informed consent. The investigation conformed with the principles outlined in the Declaration of Helsinki. The study received institutional review board approval at Cedars‐Sinai Medical Center and full informed consent was obtained from all participants prior to study participation. The original data used to support the findings of this study may be released upon application to the Women Ischemia Syndrome Evaluation Steering Committee, who can be contacted at Barbra Streisand Women's Heart Center, 310‐423‐9680. Coronary function testing was performed in women with suspected INOCA as previously described.^5^, ^13^ Coronary flow reserve (CFR) in response to intracoronary (IC) adenosine (Normal > 2.5) was assessed. CFR < 2.32, previously shown to be of prognostic significance,^1^ was also considered as a significant threshold in statistical analyses below. Coronary artery diameter response to IC nitroglycerin (∆NTG) (Normal > 20%), coronary artery diameter response to IC acetylcholine (∆Ach) (Normal > 0%), and coronary blood flow increase in to IC acetylcholine (∆CBF) (Normal > 50%) were measured.^13^ The presence of CMD was defined as a limitation in ≥1 of these tests.

### 2.2 Healthy reference control subjects

Plasma for the healthy reference control (RC) subjects (n = 50) with no self‐reported history of cardiac disease, was obtained from an ISO/FDA/USDA/EPA approved commercial source, Innovative Research (https://www.innov-research.com) with full informed consent.

### 2.3 Women with established heart failure (HFpEF and HFrEF)

Consecutive women with ambulatory HFpEF (LVEF ≥50%; n = 20) were enrolled at the time of office visit at the Cedars‐Sinai Medical Center Advanced Heart Failure Clinic between July 2014 to November 2015. As a sensitivity analysis for known myocyte dysfunction, a comparison group of women with ambulatory HFrEF (LVEF ≤40%, n = 36) was also included, also recruited from the Cedars‐Sinai Medical Center Advanced Heart Failure Clinic in the same time interval. Data collected in these cohorts included age and cardiovascular risk factors. Among the HFpEF and HFrEF women, functional capacity was measured using the maximal rate of oxygen consumption at incremental exercise (maximal VO2 was also collected), while the Duke Activity Status Inventory (DASI)^14^ estimated functional capacity measured as metabolic equivalents (METs) in the women with suspected INOCA.

### 2.4 cBIN1 assay

cBIN1 is a membrane scaffolding protein that localizes to t‐tubules and facilitates microtubule‐dependent forward delivery of calcium channels.^9^ It is a plasma biomarker associated with cardiomyocyte health,^15^, ^16^ lower levels are associated with cardiomyopathy, overt heart failure and increased arrhythmias in both animal^17^, ^18^ and human studies.^15^ As previously described, all plasma samples were collected into a lavender top (EDTA) tube and stored in the Cedars‐Sinai Medical Center Heart Institute Biobank per protocol.^11^ Immediately after being drawn, blood was stored at 4°C for less than 4 hours, centrifuged in 4°C at 2250*g* for 20 minutes, plasma supernatant was collected and aliquoted into de‐identified tubes, flash frozen, and stored at −80°C. Plasma cBIN1 concentrations were measured with a cBIN1 specific sandwich ELISA assay. The natural logarithm of plasma cBIN1's normalized reciprocal was derived, yielding a CS.

### 2.5 Statistical analysis

Descriptive analysis was conducted using means ± SD or counts and proportions. Bivariate analysis was conducted using Fisher's exact test, t‐test, or Wilcoxon rank‐sum test, as appropriate. Correlation was assessed using Spearman correlation coefficients, in order to minimize the influence of outliers. The utility of CS for predicting CMD abnormalities, using both previously defined cut‐off values^13^ was assessed using sensitivity, specificity, positive predictive value (PPV), negative predictive value (NPV) and Area Under the Curve (AUC) Receiving Operating Characteristics (ROC) analysis. A value of CS > 1.0 was used as a cutoff value for sensitivity and specificity analysis, as values of a CS > 1.0 represents one and a half standard deviations above the mean of healthy control cohorts with no known heart failure^8^, ^11^ and has been previously associated to poor‐myocyte health.^8^, ^11^ A two‐sided alpha of 0.05 was used for all tests. Comparison of CS levels in women with suspected INOCA with those in RC, HFpEF, and HFrEF groups was conducted using the Fisher's exact test, and potential confounders were assessed using Tukey adjusted pair‐wise test. Multivariable linear regression was used to compare CS levels in women with suspected INOCA with those in RC, HFpEF, and HFrEF groups. The outcome was CS and the model was adjusted for age, BMI, diabetes, and history of hypertension. An interaction of age and group was also included in the model. Functional capacity measured by VO2 max was converted to METS by dividing the maximal VO2 by 3.5 in order to allow for comparison as estimated by the DASI questionnaire.

## 3 RESULTS

Baseline characteristics are shown in Table 1. Women in the suspected INOCA, HFpEF, and HFrEF group were older than RCs (all *p* < .05), while women with HFrEF were more likely to be diabetic (*p* = .002), and women with HFpEF had higher rates of hypertension (*p* = .001). Women with suspected INOCA had higher estimated functional capacity than women with either HFpEF or HFrEF, but this did not reach statistical significance (*p* = .19).

**TABLE 1:** Comparison of cBIN1 score and baseline characteristics across different groups of women

	Reference Controls n = 50	Suspected INOCA n = 39	HFpEF n = 20	HFrEF n = 36	*p*‐value
Age (years)	53.3 (±6.2)	56.3 (±11.1)	56.7 (±12.6)	60.0 (±9.8)	**.007** ^b^
Family history of CAD	0	19 (48.7%)	4 (25.0%)	9 (30%)	.21^a^
History of smoking	0	10 (25.6%)	4 (21.1%)	3 (9.4%)	.21^a^
Hypertension	6 (12.0%)	12 (60%)	12 (60.0%)	11 (30.6%)	**.001** ^a^
Hyperlipidemia	0	9 (24.3%)	3 (20.0%)	8 (22.9%)	1.000^a^
Diabetes	4 (8.0%)	4 (10.3%)	2 (10%)	14 (38.9%)	**.002** ^a^
BMI (kg/m^2^)	30.5 (±6.8)	28.8 (±7.9)	27.9 (±7.3)	30.0 (±6.8)	.20^b^
LVEF (%)	NA	61.1 (±5.8)	61.0 (±7.6)	27.6 (±7.6)	**<.001** ^b^
CS	−0.01 (±0.7)	0.97 (±0.7)	1.7 (±0.6)	1.71 (±0.7)	**<.001** ^b^
Functional capacity (METS)^c^	NA	5.9 (±5.2)	3.8 (±0.9)	3.3 (±1.0)	.19^b^

*Note*: Data are presented as mean ± SD or n (%), bold font = *p* < .05.

Abbreviations: BMI, body mass index; CAD, coronary artery disease; CS, cBIN1 score; HFpEF, heart failure with preserved ejection fraction; HFrEF, heart failure with reduced ejection fraction; LVEF, left ventricular ejection fraction; METS, metabolic equivalents; NA, not available.

^a^ Fisher's Exact test.

^b^ Kruskal Wallis test.

^c^ Functional capacity was measured by Duke Activity Status Index questionnaire in the suspected INOCA group (METS) and cardiopulmonary testing assessed functional capacity in the HFpEF and HFrEF groups (VO2 max/3.5 = METS).

### 3.1 CS and CMD

**FIGURE 1:**
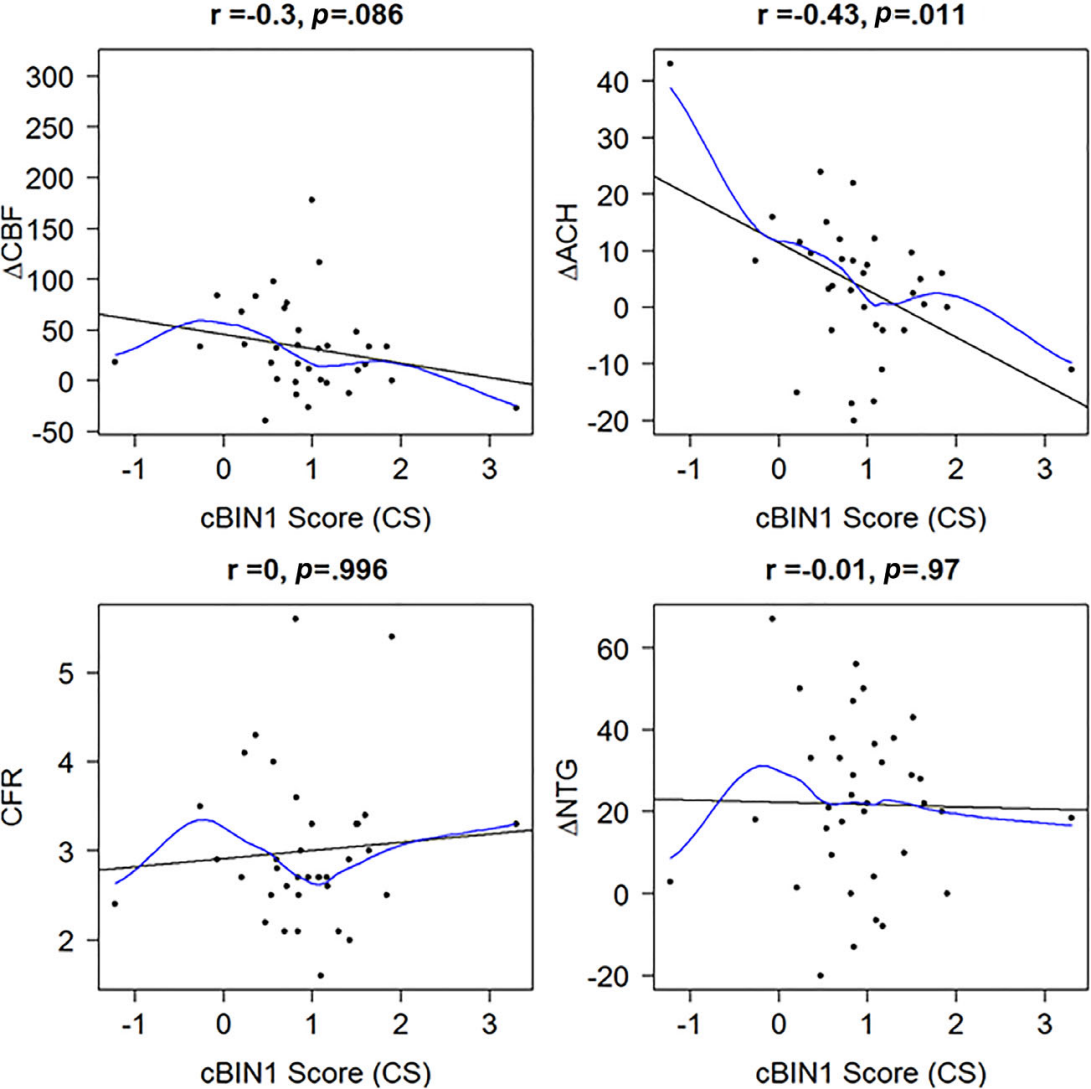
Correlation plots between cBIN1 score and invasive coronary function testing results. cBIN, cardiac bridge integrator 1; CS, cardiac binding integrator 1 score

Overall, in women with suspected INOCA, CS was ≥1.0 in 22/39 (56%). There were no differences in cardiovascular risk factor burden, age, BMI, or DASI score between women with suspected INOCA with and without CS≥1.0 (Table 2). CFR, ∆NTG, ∆CBF, and ∆Ach were lower in women with CS ≥1.0, and this difference was statistically significant for ∆Ach (−1.1 ± 8.4 vs. 6.9 ± 14.2, *p* = .047). Higher CS was moderately related to lower ΔAch (r = −0.43, *p* = .011). There was a similar nonsignificant trend for an association between Higher CS and ΔCBF (r = −0.30, *p* = .086), but not for CFR or ΔNTG (Figure 1).

**TABLE 2:** Baseline characteristics according to cBIN1 score of women with suspected INOCA

	CS ≥ 1.0 (n = 22)	CS < 1.0 (n = 17)	*p*‐value
Age (years)	54.1 ± 11.4	57.9 ± 10.9	.28^b^
Family history of CAD	8 (47.1%)	11 (50.0%)	1.00
History of smoking	2 (11.8%)	8 (36.4%)	.14
Hypertension	4 (23.5%)	8 (38.1%)	.49
Hyperlipidemia	3 (18.8%)	6 (28.6%)	.70
Diabetes	2 (11.8%)	2 (9.1%)	1.00
BMI (kg/m^2^)	27.9 ± 7.9	29.4 ± 8.1	.62^b^
DASI score (METS)	7.4 ± 6.5	4.9 ± 4.2	.36^b^
LVEDP (mmHg)	10.9 ± 4.9	10.3 ± 5.4	.76^b^
LVEF (%)	61.2 ± 6.3	61.0 ± 5.4	.88^b^
CFR, n = 36	2.9 ± 0.9	3.1 ± 0.9	.63^b^
Abnormal CFR (<2.5), n = 36	3 (20%)	4 (19.1%)	1.00
∆CBF, n = 34	21.9 ± 35.9	39.7 ± 49.3	.20^b^
Abnormal ∆CBF (<50%), n = 34	12 (92.3%)	13 (61.9%)	.107
∆Ach, n = 34	−1.1 ± 8.4	6.9 ± 14.2	**.047** ^b^
Abnormal ∆Ach (<0%), n = 34	7 (53.9%)	5 (23.8%)	.139
∆NTG, n = 36	19.1 ± 16.8	23.8 ± 22.2	.50^a^
Abnormal ∆NTG (<20%), n = 36	6 (42.9%)	9 (40.9%)	1.00
At least ≥1 abnormal CMD pathway, n = 33	14 (93.3%)	17 (77.3%)	.368

*Note*: Data are presented as mean ± SD or n (proportion). Bold font = *p* < .05.

Abbreviations: ∆Ach, change in coronary artery diameter in response to acetylcholine; BMI, body mass index; CAD, coronary artery disease; ∆CBF, change in coronary blood flow in response to acetylcholine; CFR, coronary flow reserve; CMD, coronary microvascular dysfunction; CS, cBIN1 score; DASI, Duke Activity Status Index; LVEDP, left ventricular end‐diastolic pressure; LVEF, left ventricular ejection fraction; METS, metabolic equivalents; ∆NTG, change in coronary artery diameter in response to nitroglycerin.

^a^ Tests were Fisher's Exact test or t‐test.

^b^ Wilcoxon Rank Sum test.

**FIGURE 2:**
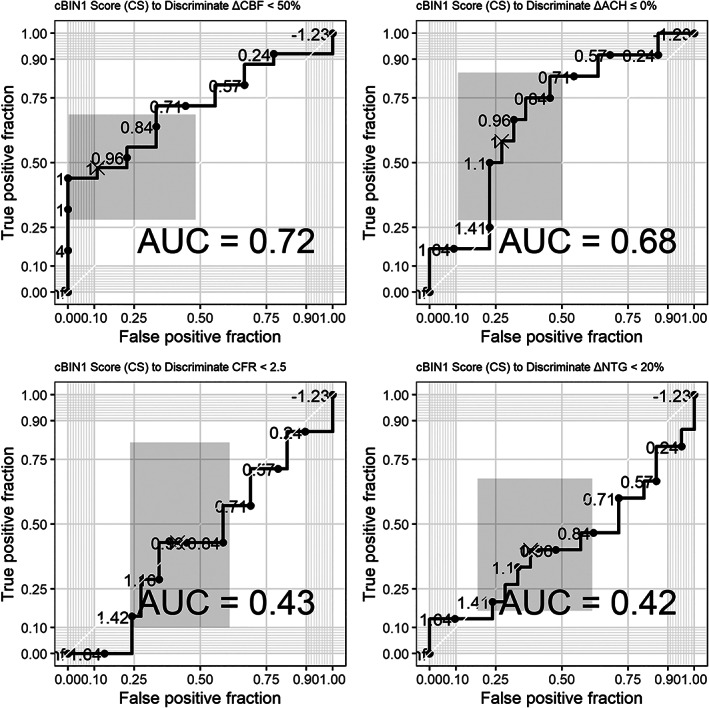
Receiver‐operator‐characteristic curves for cBIN1 score prediction of coronary microvascular dysfunction. AUC, area under the curve; cBIN, cardiac bridge integrator 1

Sensitivity and specificity analysis in women with suspected INOCA found that CS > 1.0 demonstrated high specificity (70.8%) in identifying women with abnormal ΔAch but limited sensitivity (60.0%). CS > 1.0 also demonstrated high specificity (88.9%) for abnormal ΔCBF, but limited sensitivity (48.0%). The NPV of CS was highest for women with CFR < 2.32 (85.7%). Receiver operating characteristics (ROC) analysis and area under the curve (AUC) are presented in Figure 2. CS > 1.0 discriminated between women with ∆Ach < 0% and ∆CBF < 50% (AUC 0.63 and 0.72, respectively), but was a poor discriminator for CFR < 2.5 and ∆NTG < 20% (AUC 0.43 and 0.42, respectively).

### 3.2 CS across groups

**FIGURE 3:**
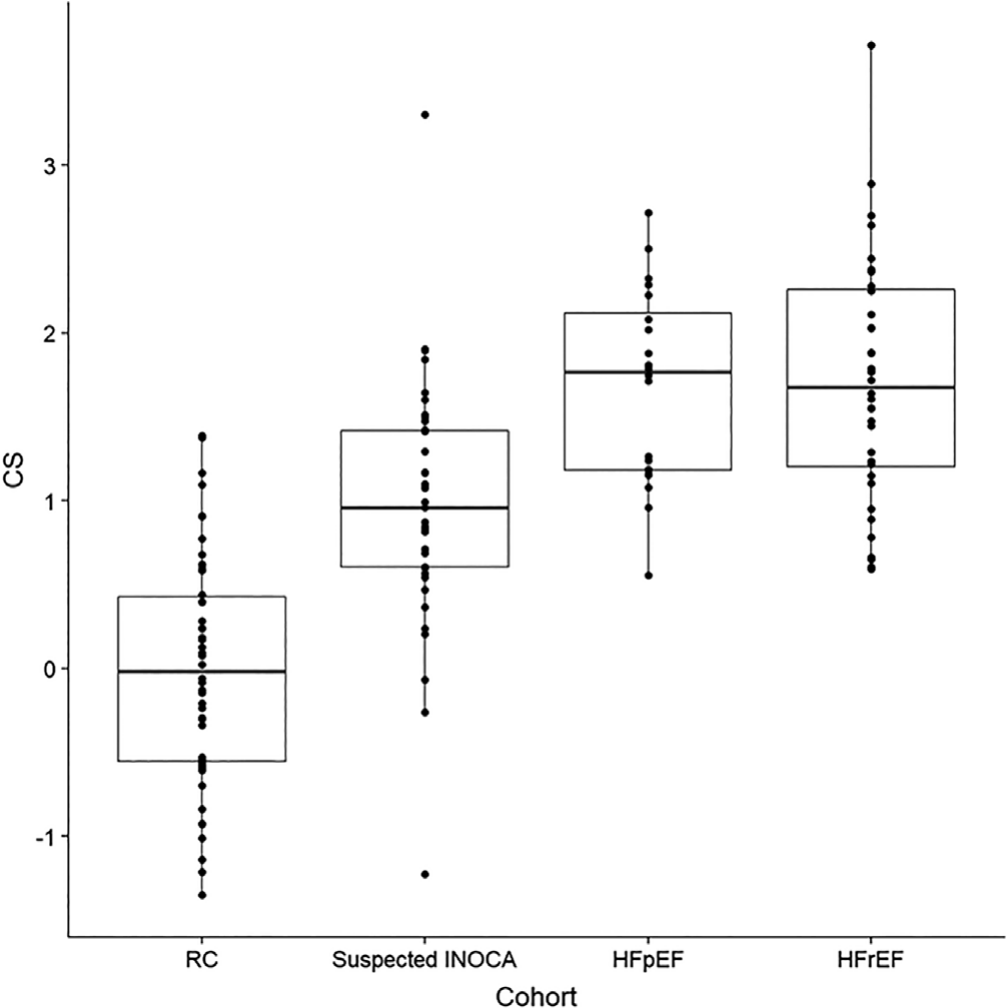
Comparison of CS levels across groups. CS, cBIN1 score; HFpEF, cohort of women with heart failure with preserved ejection fraction (n = 20); HFrEF, cohort of women with heart failure with reduced ejection fraction (n = 36); RC, reference controls with no previous history of heart disease (n = 50); suspected INOCA, invasive coronary function testing cohort (n = 39)

Comparison of mean CS in the women with suspected INOCA to RC, HFpEF and HFrEF women is shown in Figure 3. Women with suspected INOCA with CMD had CS levels significantly higher than RC women, but lower than HFpEF and HFrEF women. CS remained significantly different between groups after adjustment for age, BMI, diabetes and hypertension (Regression estimates: CRT vs. control 0.91, *p* < .01; HFpEF vs. control 1.53, *p* < .01, HFrEF vs. control 1.61, *p* < .01). Age was also independently associated to CS after adjustment for covariates (*p* = .03), and there was an interaction between age and study group, reflecting that as age increased, smaller differences in CS across groups were expected. Functional capacity was not related to CS levels within and across groups (data not shown).

## 4 DISCUSSION

CS, a plasma biomarker indicative of poor cardiomyocyte health and adverse myocardial remodeling, is associated to certain CMD pathways and may potentially be a discriminator of certain mechanisms of CMD in women with suspected INOCA. We found that CS > 1.0 demonstrated high specificity for ΔAch and CBF but limited sensitivity. CS was higher in women with suspected INOCA as compared to RC but lower than CS measured in women with either HFpEF or HFrEF. Despite the absence of obstructive coronary disease, higher CS levels in 56% of our suspected INOCA women suggests that CMD may be associated with alteration in the intracellular mechanisms responsible for appropriate myocyte function.

CS levels were independently associated to different disease groups, as was age, which may modulate differences in CS levels observed between groups. This finding contrasts with previous reports concerning the association between CS and age,^19^ may be due to the inclusion of women with a different clinical phenotype in this study, and potentially suggests that levels of CS may potentially better discriminate myocyte dysfunction in younger women, although larger studies are needed to confirm this observed interaction.

Our prior work in women with CMD demonstrates HF hospitalization is the most frequently adverse cardiovascular event at 5–7 year follow‐up,^2^ the vast majority confirmed to be HFpEF.^3^ We have hypothesized that CMD may progress to HFpEF via risk factor conditions (hypertension, dyslipidemia, dysglycemia, estrogen loss) which promote a pro‐inflammatory, pro‐oxidative state rendering the coronary microvasculature and myocardium vulnerable to: (1) adverse LV remodeling, (2) ischemia, (3) diffuse fibrosis, (4) cardiac steatosis, and (5) vascular stiffness.^20^ The current hypothesis‐generating findings suggest that CMD‐induced myocyte dysregulation may potentially contribute to subclinical myocyte dysfunction progression, potentially contributing to the development of HFpEF, which remains a heterogenous and poorly understood condition. Other evidence supports this hypothesis. Positron emission tomography (PET)‐measured CBF combined with high‐sensitivity troponin levels indicative of myocardial injury predict subsequent HFpEF.^4^ The cross‐sectional CS levels in women with suspected INOCA representing an intermediate level between the levels observed in RC as compared to women with established HF, supporting our hypothesis and suggests a prospective study may be indicated.

CMD and HFpEF share similar risk factors, with both being more common in women, and in subjects with diabetes and hypertension.^21^, ^22^ Myocytes of subjects with HFpEF demonstrate lower cyclic guanisine monophosphate (cGMP) and reduced nitric oxide activity,^23^ a characteristic, which has also been previously associated with abnormal coronary endothelial function.^21^, ^24^ Previous studies have suggested a link between coronary endothelial dysfunction, reduced NO production and the release of reactive oxygen species, which promote myocyte dysfunction via increased collagen deposition.^24^ Higher CS levels, reflecting low‐serum cBIN1, appear to be have high specificity for abnormalities in the assessment of predominantly endothelial‐dependent pathways of microvascular dysfunction, which are often assessed through evaluation of coronary artery diameter and CBF in response to acetylcholine.^13^ Endothelial dysfunction may lead to a reduction in nitric oxide, thus contributing to oxygen supply mismatch, which along with elevated left ventricular filling pressures, induces ischemia^25^ contributing to myocyte and tubular damage. These pathophysiological changes on a microcellular level, potentially reflected by increasing CS levels, may eventually leading to fibrosis, ventricular remodeling and overt HF.^25^

Higher CS suggests myocyte‐level impairment of calcium handling,^26^ which may precede development of myocyte remodeling, ventricular stiffness, increase filling pressures and a rise in brain natriuretic peptide (BNP) levels. A subgroup analysis of I‐PRESERVE, a negative randomized controlled trial of Irbesartan in patients with HFpEF, suggested an association with reduced adverse events in patients with low but not high‐BNP values, suggesting the possible existence of subclinical disease undetectable by conventional biomarkers.^27^ Higher CS has also been associated to a significantly higher rate of cardiovascular hospitalizations at 1‐year follow‐up.^11^ Early detection of an intermediate, preclinical phase of heart failure, as suggested by stratification of CS levels across our RC group, and groups of women with suspected INOCA, HFpEF, or HFrEF, should be further investigated for the noninvasive risk stratification of women with suspected INOCA for CMD detection and prediction of HFpEF.

## 5 LIMITATIONS

Limitations of our study include relatively small sample sizes; these findings are therefore hypothesis generating and subsequent evaluation in larger and prospective cohorts is needed to confirm and extend these initial findings. Our exclusive study of women undergoing clinically indicated coronary angiography reduces generalization to other women, as well as men. CMD as reflected by some of the coronary function testing results including CFR likely represent a continuum, with a range of values possibly representing abnormality or disease, since values may be modulated by extrinsic factors including heart rate and blood pressure. Although there is no strict normal dichotomic cut‐off value in the literature for these indicators of CMD, we explored association between CS levels and the most often cited cut‐off values in clinical practice and of prognostic significance. HFpEF is a heterogenous condition, with multiple etiologies, which may include CMD as a contributive underlying mechanism. The lack of BNP measurement in our cohorts precludes comparison with CS levels in this study. A standardized version of the CS assay for general use has recently been marketed. To avoid confusion in CS values, future studies should use new standardized assay.

## 6 CONCLUSIONS

CS is associated to certain CMD pathways in women with suspected INOCA, who may represent an intermediate group between healthy women and those with established HF. These findings support the hypothesis that CMD may progress to some cases of HFpEF via alteration in the intracellular processes responsible for appropriate myocyte function. Further research in larger prospective cohorts is indicated to confirm pathophysiological relations between cBIN1 levels, CMD, and HFpEF.

## Data Availability

Data sharing is not applicable to this article as no new data were created or analyzed in this study.
